# Determining the Levels of Urbanization in Iran Using Hierarchical Clustering

**Published:** 2019-06

**Authors:** Mostafa ENAYATRAD, Parvin YAVARI, Koorosh ETEMAD, Sohila KHODAKARIM, Sepideh MAHDAVI

**Affiliations:** 1. Department of Epidemiology, School of Medicine, Dezful University of Medical Sciences, Dezful, Iran; 2. Cancer Research Center, Shahid Beheshti University of Medical Sciences, Tehran, Iran; 3. Department of Health and Community Medicine, School of Medicine, Shahid Beheshti University of Medical Sciences, Tehran, Iran; 4. Department of Epidemiology, Environmental and Occupational Hazards Control Research Center, School of Public Health, Shahid Beheshti University of Medical Sciences, Tehran, Iran; 5. Department of Epidemiology, School of Public Health and Safety, Shahid Beheshti University of Medical Sciences, Tehran, Iran; 6. Department of Epidemiology, School of Public Health, Shahroud University of Medical Sciences, Shahroud, Iran

**Keywords:** Urbanization, Province, Cluster analysis, Iran

## Abstract

**Background:**

In this study, we used a variety of factors that affect urbanization in Iran to evaluate different provinces in Iran in terms of the level of urbanization.

**Methods:**

Using information from census 2011, we collected data on 33 indicators related to urbanization in 31 provinces in Iran. To rank the provinces we used density-based hierarchical clustering scheme. To determine similarities or differences between the provinces, the square of the Euclidean distance dissimilarity coefficient; Ward’s algorithm was used to merge the provinces to minimize intra-cluster variance. One-way analysis of variance (ANOVA) was used to determine the variance between the variables used to rank the provinces in terms of different levels of urbanization. Statistical analysis was performed using SPSS.

**Results:**

The provinces in Iran were combined with each other in 30 stages and classified into four levels. Taking into account the variables used to rank the level of urbanization, Tehran, and Alborz provinces were at the highest level of urbanization. On the other hand, the provinces of Sistan and Baluchistan, Kerman, North Khorasan, South Khorasan, Hormozgan, and Bushehr were at the lowest level of urbanization.

**Conclusion:**

Identification of provinces at the same level of urbanization can help us to discover the strengths and weaknesses in the infrastructures of each of them. Given the differences between various levels of urbanization, the identification of factors that are effective in the process of urbanization can help to access more information required for designing plans for the years to come.

## Introduction

During recent decades, the proportion of the urban population has increased in the world. Since the beginning of the twentieth century, the proportion of the world population in urban areas has increased from 14% to over 50% (1).

In 2010, for the first time in history the city dwellers (urban population) made up 50% of the total world population; by 2050, more than 70% of the world population will live in urban areas (2, 3).

The rapid growth of urbanization in the world was started after the industrial revolution in European countries and then it was observed in developed and developing countries. Over the past years, Iran has witnessed the rapid development of cities and an increase in urban population. Taking into account the urban population of Iran in 2006, the rate of urbanization in the mentioned year was 68.46%, which had an increasing trend as compared with 1955 (31.67%). In addition, in 2011 it reached 71.37%. According to the UN statistics, the percentage of the population living in urban areas will reach 78.2% by 2050; it indicates the continuation of this trend in the coming years (4, 5). Among the reasons for the growth of urbanization in Iran is the migration of rural population to urban areas which happens due to the income gap between these two areas and the establishment of factories and manufacturing companies in the urban centers; as a result, the chance of being employed and getting a job is higher in urban areas than in rural areas (6).

However, we are still facing some questions: what is urbanization and what is an urban area? Does urbanization only refer to a location, a concentration of population, specific physical features, or values and factors related to a specific lifestyle (7). There are different criteria for defining urban areas; each set of criteria is defined by national census offices and they differ largely in various countries. In many countries of Latin America and West Africa, an urban area is a place with 2,000 or more population, while in the United States and in Italy, respectively; an urban area must have a population of more than 5,000 and 10,000 people. Clearly, there is a vivid diversity in the criteria used for defining a city (an urban area) in the world (8).

The problem in defining the term “urban” in different countries may be due to economic and cultural differences. The differences in the definitions of “urban” observed around the world and the changes that occur over time are among the subjects of interest in comparative studies. In addition, the term “urban” does not just refer to physical mechanisms or artificial constructions, but is a state of mind too, i.e. apart from population size, which is a classification parameter; it covers other multidimensional parameters used as the classification criteria for an urban region (8). Urbanization can be defined as the expansion of a city or an increase in the population or area of a city over time. Nevertheless, there is a radical difference in the nature of urbanization between developed and developing communities because the main reason for the increase in the trend of urbanization especially in developing communities is the emigration from rural areas to cities and from small and medium towns to large cities (9, 10). Urban areas around the world are rapidly growing in terms of the size of the population and residential area (11). This growth is mainly associated with a distinct pattern known as urban sprawl, which is a social and economic process, associated with low residential density, use of separated lands, and dependency on vehicles (12). In the past, the environments of urban and rural areas were noticeably different, however because of recent progress many rural areas have experienced the factors associated with urbanization; as a consequence, the differences between city and villages are less clear (13, 14). Urbanization is under the impact of several factors and has multiple dimensions; since it is not easy and reliable to measure the variables associated with urbanization, it is necessary to use other variables as alternatives and representatives. The measurement of only one factor can result in unreliable and unstable results. Hence, it is better to integrate a number of these factors (15). In order to reduce misclassification of urban and rural areas and rank different levels of urbanization, researchers have used and examined several factors affecting people’s lifestyle in urban areas including economic and social conditions, access to training and health services, level of education, proportion of employment in economic sector, facilities available in urban are, density and population size, access to some specific facilities (such as water, electricity, gas, etc.) and access to communications tools (phone, Internet, etc.) (13, 16).

This study aimed to use a variety of factors that affect people’s lives in urban areas of Iran and evaluate the country’s provinces in terms of the level of urbanization.

## Materials and Methods

### Data and study sample

The data required for this study were extracted from the statistical yearbooks of Iran provinces based on the seventh general census of population and housing published by the Statistical Center of Iran in 2011. Concerning the national administrative divisions in 2011, Iran has 31 provinces, 400 counties, 994 districts, 1166 towns and 2507 villages with a population of over 75 million people. It has an area of more than 1.6 million square kilometers (17).

### Selection of variables

In order to rank the provinces in terms of urbanization, we evaluated a set of variables. The variables were selected based on two criteria. The first criterion was the frequent use of the variables in different studies to examine the levels of urbanization and their impact on urbanization. The second criterion was their availability at the time of the study. These variables were collected for each of the provinces separately and they covered seven groups of indices including population, human resources, communications, energy, healthcare, human capital development, and civil engineering and municipal services; these groups had 33 variables.

The study variables formed population index (such as population size, relative density of population, average household size, degree of urbanization, and annual population growth rate), human resources index (economic participation rate, unemployment rate, share of employment in agriculture, industry and services), communication index (internet penetration rate, mobile and fixed telephone penetration rate, percentage of villages with telephone lines), energy index including electricity, gas, and water (electricity consumption per thousand people, ratio of villages with electricity, gas consumption per thousand people, ratio of villages and towns with gas, and water consumption per thousand people), healthcare index (ratio of general practitioners per thousand people, ratio of nurses per thousand people, ratio of specialist per thousand people, ratio of all doctors per thousand people, and ratio of hospital beds per thousand people), Human development index (life expectancy at birth, education index, Gross Domestic Product Index) and civil engineering and municipal services (road density, rail density, proportion of public transportation services per the general population, number of vehicles being registered per population, per capita green space, and average residential area of the home).

The Human Development Index is calculated based on the geometric mean of three dimensions of life expectancy at birth, average years of education and Gross Domestic Product Index by analyzing the results of the population and housing census in 2011 for the provinces of the country (18).

### Statistical methods

In order to rank the provinces based on the stated factors, we used hierarchical clustering analysis method. In this method, the number of clusters is not known in advance and the process is either agglomerative or divisive. Indeed clustering analysis is a method for ranking regions, towns, and villages, so that places located on the same level are very similar to each other, but have significant differences with places located at other levels (19). In the agglomerative method, first every observation is placed within a separate cluster and then clusters with the highest level of similarity to each other or the least difference are integrated; this process continues and is repeated until the time when all observations fall into a cluster. In order to perform the agglomerative hierarchical clustering process, we can use different algorithms, which are different from each other in terms of their definitions, gap between two observations, and ways of formation of clusters (20).

In order to determine the similarity or difference between the provinces we used the square of the Euclidean distance dissimilarity coefficient; accordingly, the size of dissimilarity is equal to the sum of the square of differences in the values of the relevant variables. The smaller the coefficient, the provinces will be closer to each other. We used Ward’s algorithm also known as minimum variance criterion to integrate provinces so that to minimize the intra-cluster variance. Finally, the results of clustering were presented in a dendrogram chart, in which the vertical axis measures the distance between the clusters and the height of each cluster shows at which points the two clusters are merged (20). One-way analysis of variance (ANOVA) was used to determine the variance between the variables used to rank the provinces in terms of different levels of urbanization. Statistical analysis was performed using SPSS (ver. 23, Chicago, IL, USA).

## Results

**Fig. 1:**
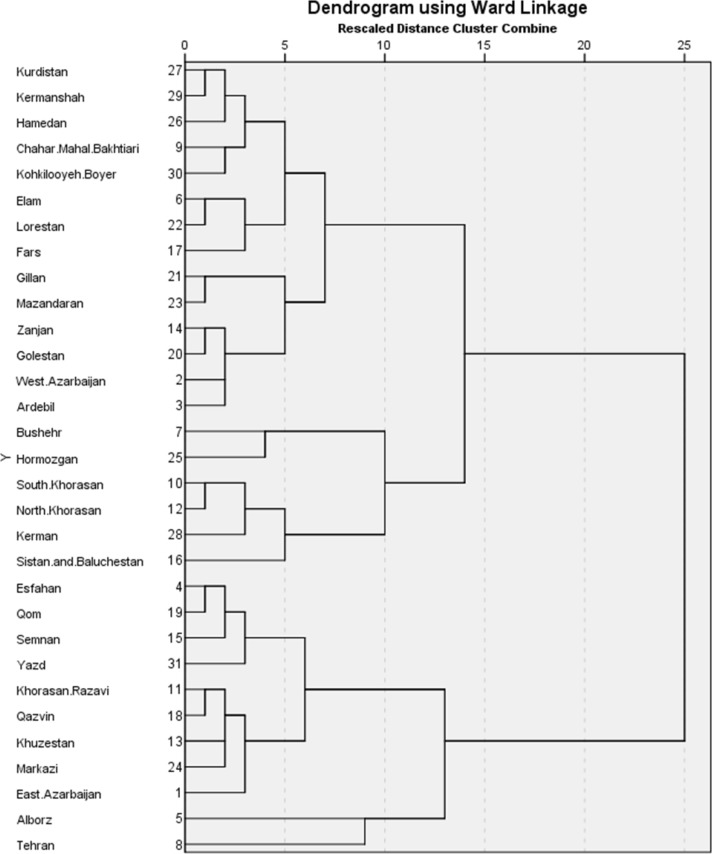
The dendrogram for levels of urbanization

The provinces were clustered in 30 stages. At each stage, two provinces with the highest level of similarity were combined with each other and placed in a cluster. The final goal of the agglomerative method is to put all the clusters in just one cluster. At the 30^th^ stage of clustering, the provinces of East Azarbaijan from the first cluster (Province NO.1 in cluster one) and West Azarbaijan (Province NO.2 in cluster two) from the second cluster were combined with a coefficient of 960 (Table 1). Dendrogram chart (Fig. 1) shows two main levels and four sub-levels of urbanization and Table 2 shows the place of the provinces in each cluster. Tehran and Alborz provinces are adjacent to each other and are placed in the first cluster. Based on the results of clustering analysis, these two provinces were at the highest level of urbanization in terms of the variables used to determine the level of urbanization. On the other hand, the provinces of Sistan and Baluchistan, Kerman, North Khorasan, South Khorasan, Hormozgan, and Bushehr were at the lowest level of urbanization.

**Table 1:** Clustering the provinces regarding urbanization

***Stage***	***Cluster Combined***		***Coefficients***	***Stage Cluster First Appears***		***Next Stage***
	***Cluster 1***	***Cluster 2***		***Cluster 1***	***Cluster 2***	
1	27	29	6.522	0	0	8
2	10	12	14.954	0	0	17
3	6	22	23.835	0	0	18
4	11	18	33.029	0	0	13
5	21	23	42.859	0	0	21
6	4	19	53.797	0	0	11
7	14	20	66.182	0	0	14
8	26	27	78.950	0	1	15
9	9	30	93.222	0	0	15
10	13	24	108.727	0	0	13
11	4	15	124.291	6	0	16
12	2	3	140.905	0	0	14
13	11	13	157.640	4	10	19
14	2	14	175.329	12	7	21
15	9	26	195.802	9	8	22
16	4	31	218.823	11	0	24
17	10	28	242.540	2	0	23
18	6	17	267.163	3	0	22
19	1	11	291.934	0	13	24
20	7	25	318.219	0	0	27
21	2	21	349.774	14	5	25
22	6	9	383.568	18	15	25
23	10	16	419.387	17	0	27
24	1	4	461.097	19	16	28
25	2	6	504.601	21	22	29
26	5	8	562.831	0	0	28
27	7	10	625.071	20	23	29
28	1	5	710.135	24	26	30
29	2	7	800.672	25	27	30
30	1	2	960.000	28	29	0

**Table 2:** Clustering provinces on urbanization

***Clustering***	***Province***
First cluster	Tehran, Alborz
Second cluster	Isfahan, Khorasan Razavi, Khuzestan, Qom, Semnan, Yazd, Qazvin, East Azerbaijan, Markazi
Third cluster	Ardebil, West Azerbaijan, Golestan, Zanjan, Mazandaran, Gilan, Fars, Lorestan, Ilam, Kohgiluyeh Boyer Ahmad, Chaharmahal and Bakhtiari, Hamedan, Kermanshah, Kurdistan
Fourth cluster	Bushehr, Hormozgan, North Khorasan, South Khorasan, Kerman, Sistan and Baluchistan

As shown in Table 3, the variables of population density (*P*<0.001), average household size (*P*=0.016), rate of urbanization (*P*<0.001), annual growth rate (*P*=0.002), unemployment rate (*P*=0.016), employment in the service sector (*P*<0.001), employment in the agricultural sector (*P*<0.001), ratio of hospital beds to population *(P*=0.024), human development index (*P*<0.001), internet penetration rate (*P*<0.001), fixed telephone penetration rate (*P*=0.002), electricity consumption rate (*P*=0.007), ratio of villages with gas to all villages in the province (*P*=0.005), ratio of cities with gas to all cities in the province (*P*<0.001), water consumption rate (*P*<0.001), per capita green space (*P*<0.001), road density (*P*=0.031), ratio of vehicles to in the city to the population in the city (*P*=0.014), ratio of vehicles registered to the population (*P*=0.014), and ratio of population in the province to the total population in the country (*P*=0.006) were at a significant level (lower than 0.05). Therefore, considering the mean values, there were significant differences between these variables at different levels of urbanization.

**Table 3:** Descriptive statistics, analysis of variance in levels of urbanization variables

***Variables***	***Mean ± Std. Deviation***					***F Statistic***	***P-Value***	***Test of Homogeneity of Variance***	
	***Level 1***	***Level 2***	***Level 3***	***Level 4***	***Total***			***Leven Statistic***	***P-Value***
Population Density	296.45 ± 680.45	31.91 ± 54.41	39.63 ± 73.90	14.04 ± 21.87	168.90 ± 97.31	56.90	0.001	49.78	0.001
Average Household Size	0.06 ± 3.30	0.022 ± 3.49	0.26 ± 3.68	0.30 ± 3.89	0.29 ± 3.64	4.08	0.016	0.948	0.431
Urbanization Rate	1.59 ± 91.66	8.48 ± 77.73	5.46 ± 60.98	7.13 ± 55.45	12.62 ± 66.75	26.21	0.001	1.478	0.243
Annual Growth	1.38 ± 2.42	0.38 ± 1.27	0.42 ± 0.85	0.87 ± 1.79	0.72 ± 1.26	6.43	0.002	6.489	0.002
Economic Partnership	1.41 ± 37	2.51 ± 36.7	3.07 ± 37.73	4.18 ± 33.95	3.28 ± 36.65	2.072	0.127	0.451	0.718
Unemployment rate	5.65 ± 15.30	2.10 ± 10.12	3.32 ± 13.65	1.41 ± 10.75	3.27 ± 12.17	4.079	0.016	2.760	0.062
Industry Employment rate	14.56 ± 52.40	5.59 ± 46.10	4.55 ± 43.21	9.86 ± 44.35	6.79 ± 44.86	1.233	0.317	5.663	0.004
Employment in the Service Sector	13.29 ± 45.30	4.08 ± 38.04	4.78 ± 29.67	3.99 ± 30.28	6.87 ± 33.22	9.544	0.001	3.314	0.035
Employment in Agriculture	1.27 ± 2.30	6.32 ± 15.83	6.46 ± 27.10	8.47 ± 25.40	9.62 ± 21.90	11.413	0.001	1.343	0.281
Ratio of Physicians to Population	0.03 ± 0.11	0.23 ± 0.29	0.28 ± 0.49	0.18 ± 0.33	0.26 ± 0.38	2.178	0.114	2.475	0.083
Ratio of Specialist to Population	0.08 ± 0.22	0.13 ± 0.26	0.05 ± 0.23	0.08 ± 0.17	0.09 ± 0.23	1.345	0.281	2.414	0.088
Ratio of Nurses to Population	0.08 ± 0.54	0.39 ± 0.78	0.52 ± 0.94	0.34 ± 0.41	0.47 ± 0.77	2.104	0.123	1.249	0.311
Ratio of Hospital beds to Population	1.04 ± 1.68	0.43 ± 1.72	0.21 ± 1.40	0.17 ± 1.14	0.39 ± 1.46	3.697	0.024	6.020	0.003
Ratio of Doctors to Population	0.06 ± 0.37	0.18 ± 0.56	0.2 ± 0.63	0.16 ± 0.40	0.2 ± 0.55	2.676	0.067	0.671	0.577
Human Development Index	0.00 ± 0.81	0.02 ± 0.74	0.02 ± 0.70	0.06 ± 0.69	0.04 ± 0.71	8.415	0.001	1.574	0.219
Internet Penetration	2.75 ± 24.05	3.43 ± 18.65	1.86 ± 14.36	4.33 ± 13.88	4.07 ± 16.14	9.573	0.001	1.763	0.178
Mobile Penetration	20.79 ± 74.38	13.90 ± 64.13	14.53 ± 57.78	12.2 ± 54.67	14.46 ± 60.13	1.316	0.290	0.218	0.883
Telephone Penetration	14.02 ± 48.06	7.25 ± 36.18	6.22 ± 30.48	4.20 ± 26.23	8.38 ± 32.45	6.686	0.002	1.573	0.219
Ration of Villages Communication	24.17 ± 82.9	10.7 ± 91.31	9.18 ± 91.89	17.09 ±82.49	12.31 ± 89.32	1.087	0.372	3.565	0.027
Electricity Consumption	0.56 ± 2.42	1.33 ± 3.46	0.45 ± 1.59	2.04 ± 3.01	1.4 ± 2.46	5.062	0.007	7.411	0.001
Villages with Electricity	24.57 ± 82.62	5.5 ± 92.45	7.22 ± 93.73	16.33 ± 83.22	10.77 ± 90.61	1.958	0.144	7.497	0.001
Gas Consumption	0.01 ± 1.96	0.69 ± 2.32	0.48 ± 1.15	3 ± 2.46	1.45 ± 1.79	1.890	0.155	4.367	0.012
Villages with Gas	15.82 ± 40	14.04 ± 31.34	18.62 ± 26.97	3.85 ± 3.03	18.4 ± 24.45	5.267	0.005	5.275	0.005
Villages with Gas	7.11 ± 92.53	10.6 ± 91.31	17.34 ± 81.17	29.41 ±26.44	29.92 ±74.25	17.315	0.001	2.587	0.074
Water Consumption	8.28 ± 79.17	9.33 ± 69.83	8.58 ± 51.64	9.04 ± 48.84	13.36 ±58.15	13.614	0.001	0.159	0.923
Average residential infrastructure	4.17 ± 88.35	16.18 ± 105.4	13.14 ± 102.33	7.48 ± 95.56	13.33 ± 101.01	1.351	0.279	2.363	0.093
Per capita green space	2.14 ± 4.29	3.95 ± 17.25	2.99 ± 5.83	10.1 ± 12.95	7.26 ± 10.42	9.852	0.001	10.645	0.000
Road Length	4.22 ± 23.73	11.04 ± 17.69	14.71 ± 31.18	9.42 ± 14.51	13.98 ±23.56	3.416	0.031	0.344	0.794
Length of Railway	4.2 ± 4.64	0.40 ± 1.27	3.08 ± 2.10	0.61 ± 0.64	2.39 ± 1.74	1.732	0.184	6.636	0.002
Urban Vehicles	1.65 ± 2.07	1.50 ±4.61	1.51 ± 3.90	1.50 ± 2.06	1.74 ± 3.63	4.272	0.014	0.049	0.985
Ratio of Vehicles to Population	22.04 ± 55.86	7.05 ± 30.99	11.15 ± 25.47	13.86 ± 30.46	13.03 ± 30	4.252	0.014	1.877	0.157
Population ratio	9.18 ± 9.69	2.70 ± 3.62	1.48 ± 2.49	0.98 ± 1.97	3.04 ± 3.18	5.200	0.006	29.240	0.001

## Discussion

Because of the development of urban areas in Iran, in recent years we have observed an increase in urban population. Consequently, the urban population increased from 31.67% in 1950 to 71.37% in 2011 and is expected to amount to 78.2% in 2050 (4, 5). In this study, we evaluated the levels of urbanization in Iran and compared the level of urbanization in different provinces. As one of the fundamental problems in the field of urban studies, there is no global standard for classifying urban environments. In fact, the use of population index to compare the difference between urban and rural area is just one of the ways used by countries for defining urban areas; nevertheless, even this definition may undergo some changes in a country over time (15).

In the present study, various components were used for ranking the provinces in terms of urbanization. It was tried to use the indices had significant impact on the process of urbanization in Iran provinces; using the selected indices we only clarified some aspects of urbanization in a specified area, however, some of the features remain hidden due to limitations in the access to data and because of drawbacks in quantitative methods. Thus, the new scale could explain the shortcomings in binary classifications (urban and rural) and sheds light on differences in the levels of urbanization between provinces that were not clear before. Various studies have used different methods to calculate the urbanization index. Daren et al. used different components to study urbanization and they showed that a multi-component scale can better show the difference between urban and rural areas and can distinguish the changes between the two environments over time (13). The multi-component scale has avoided complex statistical methods to calculate it and used the variables usually measured at the community level. Because of the simplicity of its nature, researchers use this scale to evaluate the same data. This can help to show the difference in a region over time and the differences between various regions. In addition, it can evaluate the scale components in the studied regions via similar methods (13). The provinces in the country are at four levels in terms of the level of urbanization. The urbanization level in the two provinces of Tehran and Alborz was higher than that in the other provinces of the country, thus they were located in the first cluster. The provinces of Isfahan, Khorasan Razavi, Khuzestan, Qom, Semnan, Yazd, Qazvin East Azerbaijan, and Mazandaran were located in the next cluster. The results of statistical analysis of variance between different levels of urbanization showed that the variables of population density, annual growth rate, employment in the industrial sector, employment in the service sector, ratio of hospital beds to population, ratio of proportion of villages with communication services, electricity consumption rate, ratio of villages with electricity, gas consumption rate, ratio of villages with gas, per capita green space, railway density, and population ratio were different between different levels of urbanization; thus they made significant difference between various levels of urbanization.

### Limitations

This study was conducted to determine the level of urbanization at a provincial level, thus it cannot be stated that two provinces with the same level of urbanization have cities with the equal levels of urbanization or two provinces with different levels of urbanization have cities with different level of urbanization. In this study, we determined the level of urbanization using the data published by the Statistical Center of Iran thus there might have been some other factors influencing the urbanization level not included in the collected data and not used in our study.

## Conclusion

Identification of provinces at the same level of urbanization can help us to discover the strengths and weaknesses in the infrastructures of each of them. The structural characteristics of each level of urbanization, the utilization of infrastructures and rules can help to achieve justice at national and regional levels and design appropriate policies and strategic measures. The identification of factors that are effective in the process of urbanization can help to access more information required for designing plans for the years to come.

## Ethical considerations

Ethical issues (Including plagiarism, informed consent, misconduct, data fabrication and/or falsification, double publication and/or submission, redundancy, etc.) have been completely observed by the authors.
